# Prevalence, causes and associated factors of visual impairment and blindness among older population in outreach site, Northwest Ethiopia. A dual center cross-sectional study

**DOI:** 10.4314/ahs.v23i3.79

**Published:** 2023-09

**Authors:** Melkamu Temeselew Tegegn, Aragaw Kegne Assaye, Gizachew Tilahun Belete

**Affiliations:** Department of Optometry, College of Medicine and Health Sciences, University of Gondar, Gondar, Ethiopia

**Keywords:** Visual impairment, blindness, older population, Northwest Ethiopia

## Abstract

**Purpose:**

The study aimed to determine the prevalence, causes, and associated factors of visual impairment and blindness among the older population in Outreach sites, Northwest Ethiopia.

**Methods:**

A community-outreach-based cross-sectional study was conducted on 821 older population which were selected using a systematic random sampling technique. Face-to-face interviews and ocular examinations were performed to collect the data. A binary logistic regression was performed.

**Result:**

A total of 821 participants were recruited for the study with a median age of 57 years, with a range of 40-91 years. Out of 821 participants, 41.8% had visual impairment, and 11.7% were blind. Sixty-four and fifty seven percent of visual impairment and blindness were caused by cataract. Age ≥70years (AOR=15.0; 95%CI: 8.4-26.9), rural residency (AOR=2.3; 95%CI: 1.5-3.6), non-formal education (AOR=3.0; 95%CI: 1.6-5.6), unemployed (AOR=1.5;95%CI:1.05-2.4) and history of eye examination (AOR=1.7;95%CI:1.1-2.8) were positively associated with visual impairment. Similarly, blindness was significantly associated with age ≥ 70years (AOR=7.2; 95%CI: 3.1-16.6), rural residency (AOR=2.2;95%CI:1.2-4.2), and history of eye examination (AOR=1.9; 95%CI: 1.1- 3.3).

**Conclusion:**

the prevalence of visual impairment and blindness in this study was found to be high. Age, residency, educational status, occupational status, and history of eye examination were significantly associated with visual impairment and blindness.

## Introduction

Globally in 2020, there is an estimated 295 million people to have moderate to severe visual impairment, and 43.3 million people were blind, and the number is expected to rise to 535 million by 2050 1. Besides, out of the 338.3 million visually impaired people worldwide; 55% and 71% were females and people aged >50 years, respectively 1, 2. More than 89% of visually impaired people were living in developing countries including Sub-Saharan Africa 3. In Sub-Saharan Africa, the prevalence of visual impairment and blindness were 5.3% and 1.3%, respectively 4. Moreover, the national prevalence of moderate to severe visual impairment and blindness in Ethiopia were 3.7% and 1.6%, respectively, of which more than 85 % of their causes are avoidable 5. In 2020, a global report showed that the leading causes of visual impairment were uncorrected refractive error, cataract, age-related macular degeneration (ARMD), and glaucoma, whereas the main causes of blindness were cataract, glaucoma, and uncorrected refractive error 2. In Ethiopia, cataract, uncorrected refractive error, and corneal opacity were the major causes of moderate to severe visual impairment while cataract, corneal opacity, and uncorrected refractive error were the leading causes of blindness 5.

Research showed that visual impairment and blindness affected the physical, mental and social well-being of individuals, these individuals have a risk of falls, cognitive impairment, depression, low social interaction, dependent, and are highly vulnerable to mortality 6-8. In addition, visual impairment and blindness can cause a considerable economic impact at the individual, family, and society level due to the need for the high cost of treatment, loss of productivity and income, and need for social support 9. Despite visual impairment and blindness is the major public health problem in Ethiopia; there was few and outdated evidence on the magnitude and causes of visual impairment and blindness at a community level. Thus, this study aimed to assess the prevalence, causes, and associated factors of visual impairment and blindness among the older population attending community ophthalmic services in Northwest Ethiopia. Moreover, this study will provide evidence for stakeholders to design strategies for improving an eye care service in the community and mobilize resources to reduce the burden of visual impairment and blindness.

## Materials and Methods

### Study design, Area, and Population

A community outreach-based cross-sectional study was conducted on 821 older population at Tekledingay town in Lay Armachiho district, and Chuahit town in Dembiya district in central Gondar zone, Northwest Ethiopia. According to the 2007 National Census, 157,836 population were living in the Lay Armachiho district which is located 767 kilometers far from Addis Ababa, and 271,053 population were living in Dembiya district which is located 783 kilometers far from Addis Ababa. The University of Gondar tertiary eye care and training center is the only tertiary eye care center that provides comprehensive clinical and community eye health services for eight zones and serves as a major referral center for 14 million people living in the Amhara region, Northwest Ethiopia. The department of Optometry and Ophthalmology in the University of Gondar tertiary eye care and training center is providing a continuous community ophthalmic service for the communities in the central Gondar zone in collaboration with Light for the World and other non – governmental organizations. The community ophthalmic service consists of medical therapy, cataract extraction, trichiasis surgery, and refraction with optical correction.

All population, aged > 40 years who were attending community ophthalmic services during the study period were included in the study. However, the older population with mental health and speech problems who were unable to answer the questionnaire were excluded from the study.

### Sample size determination and sampling procedure

Sample size was determined by using a single population proportion formula with an assumption of 95% confidence level, 5% marginal error, and 50% the expected proportion of visual impairment since there was no previous evidence on the magnitude of visual impairment in community ophthalmic service, the calculated sample size was 384. And then, by considering 2 design effects and a 10% of non –response rate, the final planned sample size was 845. A multistage sampling technique was employed during the sampling process. To ensure representativeness, two (25%) catchment districts were selected from 9 catchment districts in the central Gondar zone for community ophthalmic service were selected by using lottery methods. Proportionally allocation was employed to select the study participant from the selected community ophthalmic service sites.

A total of 3500 older population were attending community ophthalmic service in selected sites during the data collection period.

Due to the occurrence of Covid-19, three hundred-sixty-one (361) data were collected in Tekledingay from February 15 to 21/2020, and the remaining four hundred sixty (460) data were collected in Chuahit from June 1 to 6 /2021. The study participants were selected by applying a systematic random sampling technique.

### Operational definitions

Visual impairment and blindness were defined based on World Health Organization definitions' of visual impairment.

**Visual impairment** was defined as a presenting visual acuity of less than 6/18 in the better eye. It's further subcategorized into Moderate visual impairment was defined as a presenting visual acuity of less than 6/18 in the better eye.

**Severe visual impairment** was defined as a presenting visual acuity of less than 3/60 in the better eye, and Blindness was defined as presenting visual acuity of less than 3/60 in the better eye 10.

**Education status:** Was categorized as non-formal education (illiterate, able to write and read with no formal schooling) and formal education (from primary school to university).

**Occupational status:** Was also categorized as employed (governmental and non-governmental employer) and unemployed (having not a job, retired, housewife, and monk)

**Sunlight exposure:** participants who are exposed 5 hours and above per day were considered exposed whereas those participants who were exposed below 5 hours per day were considered non-exposed.

### Data collection procedures (personnel, instrument)

Face-to-face interviews and ocular examinations were performed to collect the data. The interview was conducted by 4 trained and senior optometrists using a pre-tested structured Amharic (local) version questionnaire, which is translated from the English version and then back to English by language translators for consistency of the tool. The questionnaire included the socio-demographic and economic data, behavioural factors, and previous ocular and medical history. History of diabetes and hypertension were assigned for the participants who had confirmed diagnosis of diabetes or hypertension previously and their respective medications are present. After completing the interview, all study participants underwent a comprehensive ocular examination. Distance visual acuity was tested in each eye using the Snellen acuity chart (tumbling E Optotypes) at 6 meters with optimal illumination. For those who could not read letters at 6 meters, their vision was measured by making the portable chart closer to the patient at specified meters up to one meter. Finally, for those who could not see any letter on the Snellen chart at 1 meter, their vision was expressed as counting fingers, hand motion, light perception with projection, and non-light perception.

Pinhole visual acuity was checked for each study participant whose distance visual acuity was less than 6/18 to exclude whether the reduction of visual acuity is due to refractive error or not.

Full refraction was performed to determine the amount and types of refractive error. Anterior segment examination was performed using a penlight torch with a 2.5X magnifier loupe. Moreover, a posterior segment examination was done using a direct Ophthalmoscope.

Regarding the cause of visual impairment and blindness, cataract was considered the main cause of visual impairment and blindness in an eye with a significant cataract, which obscured the posterior segment. Refractive error was also considered as the cause of visual impairment and blindness when the spherical equivalent (SE) was ≥ ±1.0 dioptres following full refraction. Furthermore, posterior segment diseases were considered the cause of visual impairment and blindness in cases where there was no evidence of media opacity and visual acuity did not improve with refraction. However, there was more than one cause, an ocular disease which has a profound visual reduction in the better eye and could be easily corrected or treatable was considered as the cause of visual impairment and blindness based on the suggested methodology of the World Health Organization survey on visual impairment 11.

### Data processing and analysis

After checking the completeness and consistency of data, data were coded and entered into EPI-Info version 7 and then exported into Statistical Package for Social Science (SPSS) version 20 for analysis. Descriptive statistics such as frequency and measures of central tendency were used to summarize the descriptive part of the study. A binary logistic regression was fitted to identify the possible predictors associated with visual impairment and blindness. Variables with a P-value of less than 0.2 in the bivariable analysis were entered into a multivariable binary logistic regression, and their strength of association was expressed by using an adjusted odds ratio with a 95% confidence interval. The fitness of the model was assured by using Hosmer and Lemeshow goodness of fit. Variables with a P-value of less than 0.05 were considered statistically significant.

### Ethical approval

This study was conducted in accordance with the principle of the Declaration of Helsinki and was approved by the School of Medicine Ethical Review Committee at the University of Gondar. Written informed consent was obtained from each study participant after explaining the purpose of the study. All participants were informed about their right to withdraw from the study at any time during the interview and examination. Confidentiality was maintained by avoid any personal identifier from data collection tool.

## Result

### Socio-demographic characteristics of the Study Participants

A total of 821 study participants have participated in the study, of those 59.2% were male. The median age of the study participant was 57 years, with a range of 40-91 years. In the current study, the prevalence of visual impairment was more prevalent in participants aged ≥ 70 years (49.5%), males (58.6), and living in a rural area (79.3%). Similarly, the prevalence of blindness was 64.6%, 59.4%, and 85.4% for those participants aged ≥ 70 years, males and rural dwellers, respectively (Table 1).

**Table 1:** Socio-demographic and economic data of the study participant, and disruption of presenting visual impairment and blindness (n=821)

Variables		Visual impairment (n=343)		Blindness (n=96)	
Total participants (%)		Frequency (%)	P-value	Frequency (%)	P-value
Age (years)			<0.0001		<0.0001
40-49	254(30.9)	36(10.5)		8(8.3)	
50-59	185 (22.6)	50 (14.6)		6(6.3)	
60-69	162 (19.7)	87 (25.4)		20(20.8	
≥ 70	220(26.8)	170(49.5)		62(64.6)	
**Sex**			**0.77**		**0.90**
Male	486(59.2)	201(58.6)		57(59.4)	
Female	335(40.8)	142(41.4)		39(40.6)	
**Residency**			**<0.0001**		**<0.0001**
Rural	508(61.9)	272(79.3)		82(85.4)	
Urban	313(38.1)	71(20.7)		14(14.6)	
**Current Marital status**			**<0.0001**		**0.03**
Married	665(81.0)	268 (78.1)		70(72.9)	
Divorced or widowed					
	123(15.0)	68 (19.8)		23(24.0)	
Unmarried	33(4.0)	7(2.1)		3(3.1)	
**Educational status**			**<0.0001**		**<0.0001**
Non-formal education					
	607(73.9)	319(93.0)		94(97.9)	
Formal education					
	214(26.1)	24(7.0)		2(2.1)	

**Occupational status**			**0.03**		**0.13**
Employed	527(64.2)	205(59.8)		55(57.3)	
Unemployed	294(35.8)	138(40.2)		41(42.7)	
**Monthly income (Ethiopian birr)**					
	**<0.0001**		**<0.0001**		
≤ 1000	402(49.0)	224(65.3)		72(75.0)	
1500	169(20.6)	66(19.2)		15(15.6)	
1501-					
2000	78 (9.5)	25(7.3)		5(5.2)	
≥2001	172(20.9)	28 (8.2)		4(4.2)	
**Health insurance**			**0.03**		**0.06**
Yes	288(35.1)	135(39.5)		42(43.8)	
No	533(64.9)	208(60.6)		54(56.2)	
**Living arrangement**			**0.30**		**0.07**
Living alone	85 (10.4)	40(11.7)		15(15.6)	
With family member	736(89.6)	303(88.3)		81(84.4)	

n-sample size, monthly income was categorized based on interquartile range

### Medical and ocular history, and behavioural characteristics of the study participants

Among the total study participants, 27(3.3%) were having a history of diabetes mellitus. Out of 821 study participants, 110(13.4 %) had a previous history of ocular surgery, of whom 79, 28, and 3 participants underwent cataract, trichiasis, and glaucoma surgery, respectively. Furthermore, of the total participants, only 256 (31.2%) performed an eye examination once a time in their life (Table 2).

**Table 2:** Medical, and ocular history, and behavioural characteristics of the study participants attending ophthalmic Outreach sites in Northwest Ethiopia (n=821)

Variables	Visual impairment (n=343)			Blindness (n=96)	
	Total (%)	Frequency (%)	P-value	Frequency (%)	P-value
**History of diabetes mellitus**			**0.61**		**0.19**
Yes	27 (3.3)	10(2.9)		1(1.0)	
No	794(96.7)	333(97.1)		95(99.0)	
**History of hypertension**			**0.18**		**0.48**
Yes	47(5.7)	24(7.0)		7(7.3)	
No	774(94.3)	319(93.0)		89(92.7)	
Other known systemic diseases*			**0.33**		**0.82**
Yes	38(4.6)	13(3.8)		4(4.2)	
No	783(95.4)	330(96.2)		92(95.5)	
**History of occur injury**			**0.57**		**0.73**
Yes	40(4.9)	15(4.4)		4(4.2)	
No	781(95.1)	328(95.6)		92(95.8)	
**History of ocular surgery**			**< 0.0001**		**0.004**
Yes	110(13.4)	71(20.7)		22(22.9)	
No	711(86.6)	272(79.3)		74(77.1	
**Sunlight Exposure (hours per day)**			**0.002**		**0.56**
<5	493 (60.0)	185 (53.9)		55 (57.3)	
≥5	328 (40.0)	158 (46.1)		41 (42.7)	
**Wear of eye glass**			**<0.0001**		**0.09**
Yes	49(6.0)	7(2.0)		2(2.1)	
No	772(94.0)	336 (98.0)		94 (97.9)	
**History of ocular self-medication**			**0.58**		**0.44**
Yes	46 (5.6)	21(6.1)		7(7.3)	
No	775 (94.4)	322(93.9)		89(92.7)	
**Use of traditional medication**			**0.41**		**0.24**
Yes	24 (2.9)	12 (3.5)		1(1.00)	
No	797 (97.1)	331 (96.5)		95 (99.0)	
**History of eye examination**			**<0.0001**		**0.001**
Yes	256 (31.2)	132(38.5)		44(45.8)	
No	565 (68.8)	211 (61.5)		52 (54.2)	

Note: Other known systemic diseases* included Asthma, Arteritis, Tuberculosis, HIV/AIDS, Malaria

### Prevalence of presenting aisual Impairment and blindness

In this study, the overall prevalence of visual impairment (presenting VA<6/18 in the better eye) among the older population was 41.8% (95%CI: 38.1-45.7) whereas the prevalence of presenting blindness in the better eye was 11.7 % (95%CI: 9.5-13.9) (Table-3).

**Table 3:** Level of presenting visual impairment in better eye among older population attending ophthalmic outreach sites in Northwest Ethiopia (n=821)

Level of Visual impairment	Frequency (%)	95% of a confidence interval
Normal vision	381(46.4)	42.7-49.6
Mild visual impairment	97(11.8)	9.7-14.1
Moderate visual impairment	176(21.4)	18.6-24.4
Severe visual impairment	71(8.7)	6.8-10.7
Blindness	96(11.7)	9.6-13.9

### Causes of presenting Visual impairment and blindness

This study revealed that the leading causes of visual impairment were cataract (63.8%), uncorrected refractive error (13.1%), and glaucoma (11.7%) whereas the major causes of blindness were cataract (57.3%), glaucoma (17.7%), and corneal opacity (9.4%) (Table 4).

**Table 4:** Causes of presenting visual impairment and blindness among study participants (n=343)

Ocular disorder	Level of visual impairment (VI)				Total visual impairment (%)
	Mild VI (%)	Moderate VI (%)	Severe VI (%)	Blind (%)	
Cataract	59(60.8)	115(65.4)	49(69.0)	55(57.3)	219(63.8)
Refractive error	25(25.8)	31(17.6)	9(12.7)	5(5.2)	45(13.1)
Corneal opacity	3(3.1)	12(6.8)	7(9.9)	9(9.4)	28(8.2)
Glaucoma	4(4.1)	18(10.2)	5(7.0)	17(17.7)	40(11.7)
ARMD	1(1.0)	0(0.0)	1(1.4)	8(8.3)	9(2.6)
Others*	5(5.2)	0(0.0)	0(0.0)	2(2.1)	2(0.6)
Total	97(100)	176(100)	71(100)	96(100)	343(100)

Note: ARMD-Age-related Macular Degeneration, Others* included diabetic retinopathy and posterior capsular opacification

### Age, sex, and residency specific prevalence of presenting visual impairment and blindness

The prevalence of visual impairment and blindness among female participants aged ≥70 years were 82% and 29.5%, respectively while the prevalence of visual impairment and blindness among male participants in the same age groups were 75.5% and 27.7%, respectively (Table 5).

**Table 5:** Age, Sex, and Residency specific prevalence of presenting visual impairment and blindness among study participants

Sex stratified by age and residency			Number of subject	Level of Visual Impairment (VI)				
				Mild VI	Moderate VI	Severe VI	Blind	Overall, VI
				Frequency (%)	Frequency (%)	Frequency (%)	Frequency (%)	Frequency (%)
**Male**	**Age (Years)**	40-99	113	7(6.2)	6(5.3)	1(0.9)	3(2.6)	10(8.8)
		50-99	106	10(9.4)	13(12.3)	7(6.6)	0(0)	20(18.9)
		60-69	108	15(13.9)	29(26.9)	12(11.0)	10(9.3)	51(47.2)
		≥ 70	159	22(13.8)	51(32.1)	25(15.7)	44(27.7)	120(75.5)
		**Total**	486	54(11.1)	99(20.4)	45(9.3)	57(11.7)	201(41.4)
	**Residency**	Rural	330	36(10.9)	82(24.8)	38(11.5)	51(15.5)	171(51.8)
		Urban	156	18(11.5)	17(10.9)	7(4.5)	6(3.8)	30(19.2)
		**Total**	486	54(11.1)	99(20.4)	45(9.3)	57(11.7)	201(41.4)
**Female**	**Age (Years)**	40-49	141	12(8.5)	17(12.1)	4(2.8)	5(3.5)	26(18.4)
		50-99	79	15(19.0)	20(25.3)	4(5.1)	6(7.6)	30(38.0)
		60-69	54	9(16.7)	18(33.3)	8(14.8)	10(18.5)	36(66.6)
		≥ 70	61	7(11.5)	22(36.1)	10(16.4)	18(29.5)	50(82.0)
		**Total**	335	43(12.8)	77(23.0)	26(7.8)	39(11.6)	142(42.4)
	**Residency**	Rural	178	21(11.8)	55(30.9)	15(8.4)	31(17.4)	101(56.7)
		Urban	157	22(14.0)	22(14.0)	11(7.0)	8(5.1)	41(26.1)
		**Total**	335	43(12.8)	77(23.0)	26(7.8)	39(11.6)	142(42.4)

### Factors associated with presenting visual impairment and blindness

On multivariable binary logistic regression; age, residency, educational status, occupational status, and history of eye examination were significantly associated with visual impairment

The odds of visual impairment for those participants whose ages from 60-69 years and ≥ 70 years were 5.1 times (AOR=5.1; 95%CI: 3.0-8.7) and 15 times (AOR=15.0; 95%CI: 8.4-26.9) than participants whose age 40-49 years, respectively.

Participants who were living in the rural area were 2.3 times more likely to have visually impaired than participants who were living in the urban area (AOR=2.3; 95%CI: 1.5-3.6). Participants having non–formal educational status were 3.0 times more likely to have visually impaired as compared to participants having formal educational status (AOR=3.0; 95%CI: 1.6-5.6).

This study showed that the risk of developing visual impairment was 1.5 times for unemployed participants than the employed participants (AOR=1.5; 95%CI: 1.05-2.4). As compared to participants having no history of eye examination, those participants who had a history of eye examination were 1.7 times more likely to have visually impaired (AOR=1.7;95%CI: 1.1-2.8) (Table -6)

**Table 6:** Factors associated with visual impairment among older population attending ophthalmic outreach sites in Northwest Ethiopia (n=821)

Variable	Visual Impairment				
	Yes	No	COR (95%CI)	AOR (95%CI)	P-value
**Age (years)**					**<0.0001**
40-49	36	218	1.00	1.00	
50-59	50	135	2.2(1.4-3.6)	1.9(1.2-3.2)	
60-69	87	75	7.0(4.4-11.2)	5.1(3.0-8.7)	
>70	170	50	20.6(12.8-33.0)	15.0(8.4-26.9)	
**Address**					**<0.0001**
Rural	272	236	3.9(2.9-5.4)	2.3(1.5-3.6)	
Urban	71	242	1.00	1.00	
**Current Marital status 0.11**					
Married	268	397	1.00	1.00	
Divorced or widowed	68	55	1.8(1.2-2.7)	0.8(0.32.2)	
Unmarried	7	26	0.4(0.2-0.9)	1.4(0.4-4.3)	
**Educational status**					**<0.0001**
Non-formal education	319	288	8.8(5.6-13.8)	3.0(1.6-5.6)	
Formal education	24	190	1.00	1.00	
**Occupational status**					**0.03**
Employed	205	322	1.00	1.00	
Unemployed	138	156	1.4(1.04-1.9)	1.5(1.05-2.4)	
**Monthly income (Ethiopian birr)**					**0.52**
≤1000	224	178	6.5(4.1-10.2)	0.7(0.3-1.3)	
1001-1500	66	103	3.3(2.0-5.5)	0.9(0.4-1.8)	
1501-2000	25	53	2.4(1.3-4.5)	1.0(0.4-2.1)	
≥2001	28	144	1.00	1.00	
**Health insurance**					**0.76**
Yes	135	153	1.4(1.04-1.8)	0.9(0.7-1.4)	
No	208	325	1.00	1.00	
**Wear of eyeglass**					**0.17**
Yes	7	42	1.00	1.00	
No	336	436	4.6(2.1-10.4)	2.0(0.7-5.2)	
**History of ocular surgery**					**0.13**
Yes	71	39	2.9(1.9-4.5)	0.6(0.3-1.1)	
No	272	439	1.00	1.0	
**History of eye examination**					**0.03**
Yes	132	124	1.8(1.3-2.4)	1.7(1.1-2.8)	
No	211	354	1.00	1.00	
**Sunlight Exposure (hours per day)**					**0.86**
<5	185	308	1.00	1.00	
≥ 5	158	170	1.5(1.2-2.1)	1.0(0.6-1.4)	

Regarding blindness, on applying multivariable logistic regression; age, residency, and history of eye examination were significantly associated with blindness.

Participants whose age ≥ 70 years were 7.2 times more likely to be blind than participants aged 40-49 years (AOR=7.2; 95%CI: 3.1-16.6). The risk of developing blindness in rural dwellers participants was 2.2 times (AOR=2.2; 95%CI: 1.2-4.2) than in urban dwellers participants.

Besides, participants who had a history of eye examination were 1.9 times more likely to have blind than participants who had no history of eye examination (AOR-= 1.9;95%CI:1.1-3.3) (Table 7).

**Table 7:** Factors associated with presenting blindness in better eye among older population attending ophthalmic outreach sites in Northwest Ethiopia (n=821)

Variable	Blindness		COR (95%CI)	AOR (95%CI)	P-value
	Yes	No			
**Age (years)**					**<0.0001**
40-49	8	246	1.00	1.00	
50-59	6	179	1.0(0.4-3.0)	0.8(0.3-2.4)	
60-69	20	142	4.3(1.9-10.1)	2.7(1.1-6.5)	
≥70	62	158	12.1(5.6-25.9)	7.2 (3.1-16.6)	
**Address**					**0.01**
Rural	82	426	4.1(2.3-7.4)	2.2(1.2-4.2)	
Urban	14	299	1.00	1.00	
**Monthly income (Ethiopian birr)**					**0.33**
≤1000	72	330	9.2(3.3-25.1)	2.7(0.9-8.2)	
1001-1500	15	154	4.1(1.3-12.6)	2.5(0.7-8.2)	
1501-2000	5	73	2.9(0.8-11.0)	1.9(0.5-7.6)	
≥2001	4	168	1.00	1.00	
**History of ocular surgery**					**0.20**
Yes	22	88	2.2(1.3-3.6)	0.6(0.3-1.3)	
No	74	637	1.00	1.00	
**History of eye examination**					**0.03**
Yes	44	212	2.0(1.3-3.2)	1.9(1.1-3.3)	
No	52	513	1.00	1.00	

## Discussion

In the current study, the prevalence of visual impairment was 41.8 % (95%CI: 38.1-45.7). This result is higher than institutional-based studies conducted in Debre Markos Referral Hospital Ethiopia (36.5%)^12^, Addis Ababa, Ethiopia (17.6%) 13, Ghana (31.9%)^14^, and South Africa (28.0%)^15^. This difference might be due to variation in the socio-economic characteristics of the study population, and sampling method. For example, those studies included all age groups population and used non-probability sampling. Similarly, the result of this study was also higher than the community-based studies conducted in Debre Berhan town, Ethiopia (16.8%)^16^, Gurage Zone, Ethiopia (20%)^17^, Ghana (22.7%-25.5%)^18^, ^19^, Afghanistan(22.6%)^20^, North Inia(24.5%)^21^, India(30.1%)(22), Sri Lanka(21.3%) 23, Eastern China(6.77%)^24^, and Yugan county, China (19.2%)^25^. The possible explanation for this discrepancy is that there is a variation in socio-economic characteristics of the study population, ethnicity, study setting, sampling size, and eye care service-seeking behaviours of the participants. In addition, availability, accessibility, and affordability of eye care services could also contribute to this difference.

However, the prevalence of visual impairment in this study was lower than in the studies done in South Africa(63.6)^26^, and Nepal (54.84%)^27^. The difference might be attributed due to variation in the study population in which the study participants in South Africa and Nepal were older than the participant in this study. This indicated that the burden of visual impairment can be increased as age increases because the major causes of visual impairment such as cataract, glaucoma, and age-related macular degeneration were age-related conditions.

Regarding blindness, this study showed that the prevalence of blindness was 11.7 % (95%CI: 9.6-13.9). This result is nearly agreed with other studies done in Debre Markos Referral Hospital Ethiopia(11.22%)^12^, Sudan (14%)^28^, and South Africa(10.9)^15^. In contrast, this finding is higher than the studies conducted in University of Gondar Tertiary eye care, Ethiopia(14.3%)^29^, Addis Ababa, Ethiopia (7.3%)^13^, Gurage Zone, Ethiopia(7.9%)^17^, Ghana (5.3%)^18^, Afghanistan(8.7%)^20^, North India(5%)^21^, Nepal(1.94%)^27^, Sri-Lanka(1.7)^23^, and Yugan county, China (2.27%)^25^. The difference observed here might be due to the difference in study locations, sampling size, eye care service-seeking behaviours, access to eye care service, and study population characteristics.

The current study revealed that 74.9% of visual impairment was caused by avoidable cases, of which 63.8% and 13.1% were contributed by cataract and uncorrected refractive error, respectively. Besides, cataract (57.3%) was the main cause of blindness. This finding is supported by the studies conducted in Ethiopia 5, 29 Ghana^14^, ^18^, ^19^, South Africa^15^, Afghanistan^20^, and India^22^. The possible reasons for these avoidable conditions as the leading cause of visual impairment and blindness globally particularly in developing countries might be due to low access to cataract surgery and corrective lens, social stigma for wearing spectacle, low awareness of conditions and its management, cost of treatment, availability of eye care service and waiting to maturity cataract. For instance, in this study, out of 305 cataract patients, only 79 patients underwent cataract surgery, and of 94 patients with uncorrected refractive error, 8 patients had habitual spectacle for distance vision. Thus, this finding suggested that adequate provision of cataract surgery and spectacle, and promoting public awareness about eye health through health education is required.

Furthermore, this study indicated that glaucoma was the second cause of blindness and the third cause of visual impairment which was consistent with other studies done in Ethiopia 29, Ghana^19^, South Africa^15^, and Afghanistan^20^. This might be due to the burden of glaucoma could be increased as age increases, its asymptomatic and asymmetric nature, low awareness of glaucoma, and poor seeking behaviours of eye care service. Those reasons lead to an increment of irreversible visual impairment and blindness due to glaucoma. This result implies that an extra effort will be required to create awareness about glaucoma and the importance of early vision screening. In this study, the overall prevalence of visual impairment and blindness in males was higher than in females. However, the sex-specific prevalence of visual impairment and blindness in males and females were equal and was not statistically significant. This finding is not supported by studies done in Nepal^27^ and China^24^, ^25^. This variation might be due to variation in the dominance number of participants in which those studies done in Nepal and China were dominated by female participants. This controversial finding indicated that further studies will need to show the impact of sex on the burden of visual impairment.

In the current study, the prevalence of visual impairment in those participants aged ≥70 years was 49.5% whereas the prevalence of blindness was 64.6%. The risk of developing visual impairment and blindness for participants aged ≥ 70 years was 15 .0 times and 7.2 times than participants aged from 40-49 years, respectively. This finding is consistent with other studies done in othiopia^29^, North India^21^, Nepal^27^, and China^24^, ^25^. As the age increases, the burden of age-related eye diseases such as cataract, glaucoma, and age-related macular degeneration which causes visual impairment and blindness could be increased^14^, ^27^. Moreover, an older population might have low uptake of eye care services due to poor mobility, poor systemic health, and the need for an escort. Those conditions can cause the occurrence of a high burden of visual impairment and blindness in an older population^22^.

In this study, participants who were living in the rural area were 2.3 times more likely to have visually impaired, and 2.2 times more likely to have blind than participants who were living in the urban area. This finding agreed with the study conducted in China 30. This might be due to an individual living in a rural area having poor eye care service utilization^31^.

Similarly, this study showed that participants having non–formal educational status were 3.0 times more likely to have visually impaired as compared to participants having formal educational status. This result is supported by the studies conducted in Ethiopia^16^, ^32^, Ghana 19, Afghanistan^20^, North India^21^, Nepal^27^, and China^25^. The possible justification is that individuals with low-level education had low detailed visual demands to perform the daily living activities that contribute to the delay in the uptake of eye care services. In addition, people with non-formal educational status have a poor ability to obtain outside health information, lack awareness of the disease and its management, and are more exposed to activities that are harmful to visual health^25^.

Moreover, unemployed participants were 1.5 times more likely to have visually impaired than the employed participants. The studies conducted in India^33^, and China^30^ provided similar findings. Unemployed individuals might have a low socioeconomic status which limited access to eye care services.

The current study revealed that the history of the eye examination is one of the significant factors for visual impairment and blindness, those participants who had a history of eye examination were 1.7 times more likely to have visually impaired, and 1.9 times more likely to have blind than their counterparts. This might be since eye care visits would result in the detection of more people with visual impairment. On top of that, patients who visited the eye care service regularly are patients who could have experienced serious ocular disorders that lead to more prevalent figures of visual impairment.

As a strength of the study, the study provided comprehensive evidence on visual impairment in terms of magnitude, causes and possible predictor variables. Besides, the result of the study was representative of the target population as it was conducted with appropriate methodology. As a limitation, cross –sectional nature the study did not show the exact causal relationship.

## Conclusion

The prevalence of visual impairment and blindness in this study was found to be high. Age, residency, educational status, occupational status, and history of eye examination were significantly associated with visual impairment and blindness. In the current study, cataract, uncorrected refractive error, and corneal opacity were the major causes of visual impairment whereas the leading cause of blindness was cataract, glaucoma, and corneal opacity. This finding suggested that the provision of adequate cataract surgery and spectacles, and the design of appropriate strategies for the provision of regular vision screening for populations having low socioeconomic status will require reducing the magnitude of visual impairment due to avoidable causes.

## Data Availability

The data that support the findings of this study are available from the corresponding author upon reasonable request.
